# Supplementary material for: Lysosome activity is modulated by multiple longevity pathways and is important for lifespan extension in C. elegans
Source: eLife. 2020 Jun 2;9:e55745. doi: 10.7554/eLife.55745 (PMC7274789; doi:10.7554/eLife.55745)
Supplement: Supplementary file 1. [file elife-55745-supp1.docx]

**Supplementary file 1 The 85 lysosome-related genes analyzed by RT-PCR.**

|  | **Gene** | **Description** |
| --- | --- | --- |
| **Lysosomal membrane proteins (11)** | ***scav-3*** | ortholog of human CD36 antigen |
|  | ***lmp-1*** | similar to vertebrate lysosome-associated  membrane protein CD68 |
|  | ***lmp-2*** | orthologous to LAMP  (lysosomal associated membrane glycoprotein) |
|  | ***ncr-1*** | orthologous to human NPC1 |
|  | ***ncr-2*** | orthologous to human NPC1 |
|  | ***cup-5*** | calcium transporter |
|  | ***slc-36.2*** | proton-coupled amino acid transporter |
|  | ***Y51F10.4*** | amino acid transporter |
|  | ***laat-1*** | ortholog of human PQLC2 |
|  | ***ctns-1*** | lysosomal cystine transporter |
|  | ***F13H10.3*** | ortholog of human SLC38A9 |
| **V0 subunits**  **(11)** | ***vha-1*** | ortholog of subunit c of  the membrane-bound (V0) domain of V-ATPase |
|  | ***vha-2*** | ortholog of subunit c of  the membrane-bound (V0) domain of V-ATPase |
|  | ***vha-3*** | ortholog of subunit c of  the membrane-bound (V0) domain of V-ATPase |
|  | ***vha-4*** | ortholog of subunit b of  the membrane-bound (V0) domain of V-ATPase |
|  | ***vha-5*** | ortholog of subunit a of  the membrane-bound (V0) domain of V-ATPase |
|  | ***vha-6*** | ortholog of subunit a of  the membrane-bound (V0) domain of V-ATPase |
|  | ***vha-7*** | ortholog of subunit a of  the membrane-bound (V0) domain of V-ATPase |
|  | ***vha-16*** | ortholog of subunit d of  the membrane-bound (V0) domain of V-ATPase |
|  | ***vha-17*** | ortholog of subunit e of  the membrane-bound (V0) domain of V-ATPase |
|  | ***vha-19*** | ortholog of subunit Ac45 of  the membrane-bound (V0) domain of V-ATPase |
|  | ***unc-32*** | ortholog of subunit a of  the membrane-bound (V0) domain of V-ATPase |
| **V1 subunits**  **(9)** | ***vha-8*** | ortholog of subunit E of  the cytoplasmic (V1) domain of V-ATPase |
|  | ***vha-9*** | ortholog of subunit F of  the cytoplasmic (V1) domain of V-ATPase |
|  | ***vha-10*** | ortholog of subunit G of  the cytoplasmic (V1) domain of V-ATPase |
|  |  |  |
|  | ***vha-11*** | ortholog of subunit C of  the cytoplasmic (V1) domain of V-ATPase |
|  | ***vha-12*** | ortholog of subunit B of  the cytoplasmic (V1) domain of V-ATPase |
|  | ***vha-13*** | ortholog of subunit A of  the cytoplasmic (V1) domain of V-ATPase |
|  | ***vha-14*** | ortholog of subunit D of  the cytoplasmic (V1) domain of V-ATPase |
|  | ***vha-15*** | ortholog of subunit H of  the cytoplasmic (V1) domain of V-ATPase |
|  | ***vha-18*** | ortholog of subunit H of  the cytoplasmic (V1) domain of V-ATPase |
| **Protease (cathepsins)**  **(26)** | ***ctsa-1*** | ortholog of human CTSA (cathepsin A) |
|  | ***Y40D12A.2*** | ortholog of human CTSA (cathepsin A) |
|  | ***F41C3.5*** | ortholog of human CTSA (cathepsin A) |
|  | ***K10C2.1*** | ortholog of human CTSA (cathepsin A) |
|  | ***Y16B4A.2*** | ortholog of human CTSA (cathepsin A) |
|  | ***F32A5.3*** | ortholog of human CTSA (cathepsin A) |
|  | ***K10B2.2*** | ortholog of human CTSA (cathepsin A) |
|  | ***asp-1*** | ortholog of cathepsin D aspartic protease |
|  | ***asp-3*** | ortholog of aspartyl protease |
|  | ***asp-4*** | ortholog of aspartyl protease |
|  | ***asp-8*** | ortholog of aspartyl protease |
|  | ***asp-13*** | ortholog of aspartyl protease |
|  | ***cpr-1*** | ortholog of human CTSB (cathepsin B) |
|  | ***cpr-2*** | ortholog of human CTSB (cathepsin B) |
|  | ***cpr-3*** | ortholog of human CTSB (cathepsin B) |
|  | ***cpr-4*** | ortholog of human CTSB (cathepsin B) |
|  | ***cpr-5*** | ortholog of human CTSB (cathepsin B) |
|  | ***cpr-6*** | ortholog of human CTSB (cathepsin B) |
|  | ***cpr-8*** | ortholog of human CTSB (cathepsin B) |
|  | ***T28H10.3*** | ortholog of human CTSB (cathepsin B) |
|  | ***cpl-1*** | member of the cathepsin L-like cysteine protease family |
|  | ***cpz-1*** | ortholog of cathepsin z-like cysteine protease |
|  | ***tag-196*** | ortholog of human CTSF |
|  | ***tag-329*** | ortholog of human CTSO |
|  | ***Y40H7A.10*** | ortholog of human CTSO |
|  | ***F15D4.4*** | cysteine-type peptidase |
| **Non-protease hydrolases**  **(28)** | ***asm-1*** | sphingomyelin phosphodiesterase |
|  | ***asm-2*** | sphingomyelin phosphodiesterase |
|  | ***asm-3*** | sphingomyelin phosphodiesterase |
|  | ***sul-1*** | the sole *C. elegans* 6-O-endosulfatase |
|  | ***sul-2*** | orthologous to the human gene encoding arylsulfatase A |
|  | ***sul-3*** | orthologous to the human gene encoding arylsulfatase B |
|  | ***hex-1*** | a beta-N-acetylhexosaminidase |
|  | ***hex-2*** | a beta-N-acetylhexosaminidase |
|  | ***hex-3*** | a beta-N-acetylhexosaminidase |
|  | ***hex-4*** | a beta-N-acetylhexosaminidase |
|  | ***hex-5*** | a beta-N-acetylhexosaminidase |
|  | ***gba-1*** | homologous to the human gene encoding glucocerebrosidase |
|  | ***gba-2*** | homologous to the human gene encoding glucocerebrosidase |
|  | ***gba-3*** | homologous to the human gene encoding glucocerebrosidase |
|  | ***gba-4*** | homologous to the human gene encoding glucocerebrosidase |
|  | ***pho-1*** | acid phosphatase |
|  | ***pho-10*** | acid phosphatase |
|  | ***Y105E8B.9*** | orthologous to the human gene encoding glutathione transferase zeta 1 |
|  | ***M05B5.4*** | phospholipase A2 |
|  | ***rnst-2*** | ribonuclease |
|  | ***lipl-1*** | orthologous to members of the human Lipase family |
|  | ***lipl-2*** | orthologous to members of the human Lipase family |
|  | ***lipl-3*** | orthologous to members of the human Lipase family |
|  | ***lipl-4*** | orthologous to members of the human Lipase family |
|  | ***lipl-5*** | orthologous to members of the human Lipase family |
|  | ***lipl-6*** | orthologous to members of the human Lipase family |
|  | ***lipl-7*** | orthologous to members of the human Lipase family |
|  | ***lipl-8*** | orthologous to members of the human Lipase family |
