# Supplementary material for: Lysosome activity is modulated by multiple longevity pathways and is important for lifespan extension in C. elegans
Source: eLife. 2020 Jun 2;9:e55745. doi: 10.7554/eLife.55745 (PMC7274789; doi:10.7554/eLife.55745)
Supplement: Supplementary file 2. [file elife-55745-supp2.docx]

**Supplementary file 2 Expression of 43 lysosomal genes is reduced in wild type (WT) at Day 5.**

|  | **Gene** | **Relative mRNA level in WT^a^**  **(Day 5 vs Day 1)** | | | **Mean** | **S.D.** |
| --- | --- | --- | --- | --- | --- | --- |
| **Lysosomal membrane proteins (3)** | ***lmp-2*** | 0.46 | 0.65 | 0.49 | 0.53 | 0.10 |
|  | ***ncr-1*** | 0.73 | 0.65 | 0.54 | 0.64 | 0.09 |
|  | ***slc-36.2*** | 0.45 | 0.52 | 0.71 | 0.56 | 0.13 |
| **V0 subunits**  **(8)** | ***vha-2*** | 0.56 | 0.61 | 0.61 | 0.60 | 0.03 |
|  | ***vha-3*** | 0.28 | 0.23 | 0.57 | 0.36 | 0.18 |
|  | ***vha-4*** | 0.66 | 0.56 | 0.83 | 0.68 | 0.13 |
|  | ***vha-5*** | 0.17 | 0.27 | 0.29 | 0.24 | 0.07 |
|  | ***vha-6*** | 0.30 | 0.34 | 0.31 | 0.31 | 0.02 |
|  | ***vha-16*** | 0.40 | 0.59 | 0.85 | 0.61 | 0.22 |
|  | ***vha-17*** | 0.81 | 0.65 | 0.66 | 0.71 | 0.09 |
|  | ***vha-19*** | 0.38 | 0.77 | 0.37 | 0.51 | 0.23 |
| **V1 subunits**  **(7)** | ***vha-8*** | 0.45 | 0.39 | 0.60 | 0.48 | 0.11 |
|  | ***vha-10*** | 0.22 | 0.55 | 0.64 | 0.47 | 0.22 |
|  | ***vha-11*** | 0.22 | 0.40 | 0.55 | 0.39 | 0.17 |
|  | ***vha-12*** | 0.05 | 0.31 | 0.50 | 0.28 | 0.23 |
|  | ***vha-13*** | 0.63 | 0.36 | 0.35 | 0.45 | 0.16 |
|  | ***vha-14*** | 0.45 | 0.44 | 0.73 | 0.54 | 0.17 |
|  | ***vha-15*** | 0.36 | 0.29 | 0.51 | 0.39 | 0.11 |
| **Protease (cathepsins)**  **(17)** | ***ctsa-1*** | 0.30 | 0.13 | 0.44 | 0.29 | 0.15 |
|  | ***Y40D12A.2*** | 0.19 | 0.33 | 0.53 | 0.35 | 0.17 |
|  | ***K10C2.1*** | 0.72 | 0.41 | 0.75 | 0.62 | 0.19 |
|  | ***Y16B4A.2*** | 0.50 | 0.32 | 0.42 | 0.41 | 0.09 |
|  | ***F32A5.3*** | 0.27 | 0.28 | 0.53 | 0.36 | 0.15 |
|  | ***K10B2.2*** | 0.32 | 0.28 | 0.32 | 0.31 | 0.02 |
|  | ***asp-1*** | 0.46 | 0.22 | 0.28 | 0.32 | 0.12 |
|  | ***asp-3*** | 0.34 | 0.59 | 0.52 | 0.48 | 0.13 |
|  | ***asp-4*** | 0.63 | 0.38 | 0.46 | 0.49 | 0.13 |
|  | ***asp-8*** | 0.65 | 0.83 | 0.26 | 0.58 | 0.29 |
|  | ***cpr-5*** | 0.14 | 0.15 | 0.26 | 0.18 | 0.07 |
|  | ***cpr-6*** | 0.31 | 0.33 | 0.44 | 0.36 | 0.07 |
|  | ***cpr-8*** | 0.49 | 0.36 | 0.12 | 0.32 | 0.19 |
|  | ***cpl-1*** | 0.34 | 0.33 | 0.35 | 0.34 | 0.01 |
|  | ***cpz-1*** | 0.25 | 0.32 | 0.26 | 0.28 | 0.04 |
|  | ***tag-196*** | 0.35 | 0.40 | 0.76 | 0.50 | 0.23 |
|  | ***Y40H7A.10*** | 0.73 | 0.48 | 0.67 | 0.62 | 0.13 |
| **Non-protease**  **hydrolases**  **(8)** | ***asm-1*** | 0.64 | 0.40 | 0.49 | 0.51 | 0.12 |
|  | ***sul-3*** | 0.57 | 0.36 | 0.42 | 0.45 | 0.11 |
|  | ***hex-5*** | 0.26 | 0.39 | 0.25 | 0.30 | 0.08 |
|  | ***gba-3*** | 0.14 | 0.13 | 0.60 | 0.29 | 0.27 |
|  | ***gba-4*** | 0.23 | 0.40 | 0.55 | 0.39 | 0.16 |
|  | ***Y105E8B.9*** | 0.17 | 0.16 | 0.18 | 0.17 | 0.01 |
|  | ***pho-1*** | 0.11 | 0.37 | 0.27 | 0.25 | 0.13 |
|  | ***lipl-7*** | 0.43 | 0.49 | 0.38 | 0.44 | 0.06 |

^a^Quantitative RT-PCR was performed and data were analyzed as described in the Materials and methods.
