# Supplementary material for: Lysosome activity is modulated by multiple longevity pathways and is important for lifespan extension in C. elegans
Source: eLife. 2020 Jun 2;9:e55745. doi: 10.7554/eLife.55745 (PMC7274789; doi:10.7554/eLife.55745)
Supplement: Supplementary file 3. [file elife-55745-supp3.docx]

**Supplementary file 3 Expression of 13 lysosomal genes is increased in wild type (WT) at Day 5.**

|  | **Gene** | **Relative mRNA level in WT (Day 5 vs Day 1)^a^** | | | **Mean** | **S.D.** |
| --- | --- | --- | --- | --- | --- | --- |
| **Lysosomal membrane proteins (2)** | ***Y51F10.4*** | 1.20 | 1.51 | 1.46 | 1.39 | 0.17 |
|  | ***ctns-1*** | 1.29 | 1.41 | 1.33 | 1.34 | 0.07 |
| **Protease (cathepsins)**  **(4)** | ***cpr-2*** | 5.64 | 10.23 | 11.37 | 9.08 | 3.04 |
|  | ***cpr-4*** | 4.48 | 6.03 | 5.22 | 5.24 | 0.77 |
|  | ***T28H10.3*** | 8.31 | 9.79 | 3.35 | 7.15 | 3.37 |
|  | ***tag-329*** | 1.29 | 1.35 | 2.85 | 1.83 | 0.89 |
| **Non-protease hydrolases**  **(7)** | ***lipl-1*** | 2.16 | 2.45 | 2.38 | 2.33 | 0.15 |
|  | ***lipl-3*** | 3.99 | 3.83 | 2.37 | 3.40 | 0.90 |
|  | ***asm-3*** | 0.69 | 2.96 | 3.35 | 2.33 | 1.44 |
|  | ***hex-2*** | 1.30 | 1.33 | 2.85 | 1.83 | 0.89 |
|  | ***hex-3*** | 1.19 | 2.32 | 1.32 | 1.61 | 0.62 |
|  | ***gba-1*** | 6.28 | 1.98 | 2.02 | 3.43 | 2.47 |
|  | ***gba-2*** | 3.42 | 9.93 | 3.01 | 5.45 | 3.88 |

^a^Quantitative RT-PCR was performed and data were analyzed as described in the Materials and methods.
