# Supplementary material for: Lysosome activity is modulated by multiple longevity pathways and is important for lifespan extension in C. elegans
Source: eLife. 2020 Jun 2;9:e55745. doi: 10.7554/eLife.55745 (PMC7274789; doi:10.7554/eLife.55745)
Supplement: Supplementary file 4. [file elife-55745-supp4.docx]

**Supplementary file 4 Expression of 29 lysosome genes is unaltered in wild type (WT) at Day 5.**

|  | **Gene** | **Relative mRNA level in WT (Day 5 vs Day 1)^a^** | | | **Mean** | **S.D.** |
| --- | --- | --- | --- | --- | --- | --- |
| **Lysosomal membrane proteins**  **(6)** | ***scav-3*** | 0.75 | 0.54 | 0.68 | 0.66 | 0.10 |
|  | ***lmp-1*** | 0.62 | 0.90 | 0.85 | 0.79 | 0.15 |
|  | ***ncr-2*** | 1.02 | 0.98 | 2.09 | 1.36 | 0.63 |
|  | ***cup-5*** | 1.56 | 1.15 | 0.40 | 1.04 | 0.59 |
|  | ***laat-1*** | 0.86 | 1.41 | 0.59 | 0.95 | 0.42 |
|  | ***F13H10.3*** | 0.96 | 0.88 | 1.40 | 1.08 | 0.28 |
| **V0 subunits**  **(3)** | ***vha-1*** | 0.86 | 0.77 | 1.11 | 0.91 | 0.18 |
|  | ***vha-7*** | 1.18 | 1.08 | 0.91 | 1.06 | 0.13 |
|  | ***unc-32*** | 0.71 | 0.91 | 1.02 | 0.88 | 0.16 |
| **V1 subunits**  **(2)** | ***vha-9*** | 0.94 | 0.81 | 1.22 | 0.99 | 0.21 |
|  | ***vha-18*** | 1.10 | 0.93 | 0.75 | 0.93 | 0.18 |
| **Protease (cathepsins)**  **(5)** | ***F41C3.5*** | 0.87 | 0.79 | 0.74 | 0.80 | 0.06 |
|  | ***asp-13*** | 1.35 | 1.13 | 0.77 | 1.08 | 0.29 |
|  | ***cpr-1*** | 0.81 | 0.76 | 0.58 | 0.71 | 0.12 |
|  | ***cpr-3*** | 0.73 | 1.30 | 1.74 | 1.26 | 0.51 |
|  | ***F15D4.4*** | 0.83 | 1.16 | 1.08 | 1.02 | 0.17 |
| **Non-protease hydrolases**  **(13)** | ***asm-2*** | 0.80 | 1.71 | 0.85 | 1.12 | 0.51 |
|  | ***sul-1*** | 0.69 | 0.37 | 1.65 | 0.90 | 0.66 |
|  | ***sul-2*** | 0.75 | 0.62 | 1.04 | 0.80 | 0.21 |
|  | ***hex-1*** | 1.11 | 1.04 | 0.89 | 1.01 | 0.11 |
|  | ***hex-4*** | 0.81 | 0.78 | 1.21 | 0.93 | 0.24 |
|  | ***pho-10*** | 0.99 | 0.92 | 0.81 | 0.91 | 0.09 |
|  | ***M05B5.4*** | 0.62 | 0.84 | 0.96 | 0.80 | 0.17 |
|  | ***rnst-2*** | 1.03 | 1.19 | 1.13 | 1.12 | 0.08 |
|  | ***lipl-2*** | 0.95 | 1.62 | 1.52 | 1.36 | 0.36 |
|  | ***lipl-4*** | 0.82 | 1.38 | 1.13 | 1.11 | 0.28 |
|  | ***lipl-5*** | 0.67 | 0.59 | 1.42 | 0.89 | 0.45 |
|  | ***lipl-6*** | 0.63 | 1.04 | 0.80 | 0.82 | 0.21 |
|  | ***lipl-8*** | 2.05 | 1.07 | 1.17 | 1.43 | 0.54 |

^a^Quantitative RT-PCR was performed and data were analyzed as described in the Materials and methods.
