# Supplementary material for: Lysosome activity is modulated by multiple longevity pathways and is important for lifespan extension in C. elegans
Source: eLife. 2020 Jun 2;9:e55745. doi: 10.7554/eLife.55745 (PMC7274789; doi:10.7554/eLife.55745)
Supplement: Supplementary file 5. [file elife-55745-supp5.docx]

**Supplementary file 5 Lysosome gene expression is upregulated in *daf-2*, *eat-2* and *isp-1* mutants*.***

| **Lysosomal genes with upregulated expression in *daf-2(e1370)* mutants (Day 1)** | | | | | |
| --- | --- | --- | --- | --- | --- |
| **Lysosomal gene** | **Relative mRNA level**  **(*daf-2* vs WT)^a^** | | | **Mean** | **S.D.** |
| ***lmp-2*** | 2.58 | 2.87 | 3.78 | 3.08 | 0.63 |
| ***slc-36.2*** | 3.51 | 1.50 | 3.17 | 2.73 | 1.07 |
| ***ncr-1*** | 5.10 | 4.59 | 3.22 | 4.31 | 0.97 |
| ***vha-5*** | 2.52 | 1.84 | 2.56 | 2.31 | 0.41 |
| ***vha-10*** | 3.50 | 2.50 | 3.08 | 3.03 | 0.50 |
| ***vha-14*** | 2.01 | 2.17 | 2.94 | 2.37 | 0.50 |
| ***ctsa-1*** | 3.47 | 5.43 | 6.07 | 4.99 | 1.35 |
| ***Y40D12A.2*** | 2.31 | 2.53 | 2.28 | 2.37 | 0.14 |
| ***Y16B4A.2*** | 1.88 | 1.92 | 2.19 | 2.00 | 0.17 |
| ***K10B2.2*** | 1.73 | 2.13 | 2.38 | 2.08 | 0.33 |
| ***asp-3*** | 1.48 | 3.75 | 3.18 | 2.80 | 1.18 |
| ***asp-4*** | 3.66 | 2.42 | 4.38 | 3.49 | 0.99 |
| ***asp-8*** | 4.35 | 3.48 | 8.57 | 5.47 | 2.72 |
| ***tag-196*** | 3.36 | 2.73 | 3.03 | 3.04 | 0.31 |
| ***gba-3*** | 5.69 | 2.70 | 5.28 | 4.56 | 1.62 |
| ***hex-5*** | 2.71 | 3.55 | 3.86 | 3.37 | 0.59 |
| ***Y105E8B.9*** | 2.61 | 2.94 | 2.15 | 2.56 | 0.40 |
| ***asm-1*** | 1.76 | 1.79 | 1.79 | 1.78 | 0.01 |
| ***sul-3*** | 1.42 | 2.51 | 3.63 | 2.52 | 1.10 |
| ***lipl-7*** | 2.97 | 3.57 | 1.54 | 2.70 | 1.04 |

| **Lysosomal genes with upregulated expression in *eat-2(ad1116)* mutants (Day 1)** | | | | | |
| --- | --- | --- | --- | --- | --- |
| **Lysosomal gene** | **Relative mRNA level**  **(*eat-2* vs WT)^a^** | | | **Mean** | **S.D.** |
| ***lmp-2*** | 5.32 | 2.20 | 3.36 | 3.63 | 1.58 |
| ***slc-36.2*** | 1.66 | 2.10 | 2.03 | 1.93 | 0.24 |
| ***ncr-1*** | 1.64 | 2.13 | 3.03 | 2.26 | 0.70 |
| ***vha-2*** | 1.67 | 2.25 | 1.80 | 1.91 | 0.31 |
| ***vha-3*** | 2.46 | 1.93 | 3.13 | 2.50 | 0.60 |
| ***vha-4*** | 2.56 | 1.40 | 2.42 | 2.12 | 0.63 |
| ***vha-6*** | 3.84 | 1.29 | 2.69 | 2.60 | 1.28 |
| ***vha-19*** | 1.95 | 1.80 | 2.10 | 1.95 | 0.15 |
| ***vha-8*** | 2.31 | 2.44 | 3.41 | 2.72 | 0.60 |
| ***vha-12*** | 1.31 | 1.96 | 2.62 | 1.96 | 0.66 |
| ***vha-15*** | 2.55 | 2.09 | 2.95 | 2.53 | 0.43 |
| ***Y105E8B.9*** | 3.19 | 6.28 | 5.30 | 4.93 | 1.58 |
| ***pho-1*** | 2.15 | 5.87 | 2.74 | 3.59 | 2.00 |
| ***lipl-7*** | 6.26 | 12.77 | 4.43 | 7.82 | 4.38 |

| **Lysosomal genes with upregulated expression in *isp-1(qm150)* mutants (Day 1)** | | | | | |
| --- | --- | --- | --- | --- | --- |
| **Lysosomal gene** | **Relative mRNA level**  **(*isp-1* vs WT)^a^** | | | **Mean** | **S.D.** |
| ***vha-8*** | 2.91 | 4.40 | 2.89 | 3.40 | 0.87 |
| ***ctsa-1*** | 11.50 | 15.99 | 10.54 | 12.68 | 2.91 |
| ***Y40D12A.2*** | 6.61 | 11.19 | 5.39 | 7.73 | 3.06 |
| ***asp-4*** | 3.78 | 2.23 | 1.23 | 2.41 | 1.29 |
| ***asp-8*** | 3.56 | 3.32 | 5.95 | 4.28 | 1.45 |
| ***cpr-8*** | 5.50 | 1.93 | 2.97 | 3.47 | 1.84 |
| ***asm-1*** | 2.15 | 2.73 | 2.98 | 2.62 | 0.42 |
| ***sul-3*** | 3.08 | 5.29 | 2.48 | 3.62 | 1.48 |
| ***Y105E8B.9*** | 3.31 | 5.55 | 2.91 | 3.92 | 1.43 |
| ***lipl-7*** | 2.63 | 2.38 | 4.16 | 3.05 | 0.97 |

^a^Quantitative RT-PCR was performed and data were analyzed as described in the Materials and methods.
