# Supplementary material for: Lysosome activity is modulated by multiple longevity pathways and is important for lifespan extension in C. elegans
Source: eLife. 2020 Jun 2;9:e55745. doi: 10.7554/eLife.55745 (PMC7274789; doi:10.7554/eLife.55745)
Supplement: Supplementary file 6. [file elife-55745-supp6.docx]

**Supplementary file 6 Primers used for quantitative RT-PCR, related to key resource table.**

| **Primer** | **Sequences (5’ to 3’)** | **Identifier** |
| --- | --- | --- |
| *scav-3* qPCR S | CGCAGAATCGAGCATTTTGT | PYL27 |
| *scav-3* qPCR AS | GGCATCGTGACAAGTCGATA | PYL28 |
| *lmp-1* qPCR S | GTTCACGGTCACATTCAACG | PDFZ929 |
| *lmp-1* qPCR AS | TAACGGGCAGATTGTCCTTC | PDFZ930 |
| *lmp-2* qPCR S | ACAACAAAAGAGCCGTGTAT | PYNS445 |
| *lmp-2* qPCR AS | TCGTAACGTTGCATGATGACA | PYNS446 |
| *ncr-1* qPCR S | AGGCCATGGAAGTTCAGAGA | PDFZ939 |
| *ncr-1* qPCR AS | GCTGGCGTCCAGTAAAGAAG | PDFZ940 |
| *ncr-2* qPCR S | GGGAACGAAGAATCTTGCAC | PDFZ979 |
| *ncr-2* qPCR AS | CCAGCTGATTTGTGACATGG | PDFZ980 |
| *ctns-1* qPCR S | ATGGCTGTAACCTGCTGTAA | PYNS350 |
| *ctns-1* qPCR AS | AGAATATCGAGAGTTCCTCC | PYNS351 |
| *F13H10.3* qPCR S | GGCCTGGAGCGATTAGAGTT | PDFZ933 |
| *F13H10.3* qPCR AS | TGCAGGAAGGGCAAATACAT | PDFZ934 |
| *laat-1* qPCR S | AGACGCCAACTGTACCCAAG | PDFZ931 |
| *laat-1* qPCR AS | GGAATGAGCCAAAGAGCAAG | PDFZ932 |
| *slc-36.2* qPCR S | TAGTGCTTTGTGCACAATTCG | PYNS590 |
| *slc-36.2* qPCR AS | TGGAAGGGAGTCCAGTGGTT | PYNS591 |
| *Y51F10.4* qPCR S | ACCGCTTATGATGTTCCCGG | PYNS592 |
| *Y51F10.4* qPCR AS | AATTACGGCTGTCAGAATGT | PYNS593 |
| *cup-5* qPCR S | CAACGGTGACGATATGTTCG | PDFZ937 |
| *cup-5* qPCR AS | TCATATGCATCCATGATAATTGC | PDFZ938 |
| *C08H9.1(ctsa-1)* qPCR S | AGGGATTGATAGAGCATGGAAAGATAGGA | PYNS415 |
| *C08H9.1(ctsa-1)* qPCR AS | TCGGAACGCAGGACCCGTAAA | PYNS416 |
| *Y40D12A.2* qPCR S | TCGCTGAGAATTATCAATCG | PYNS571 |
| *Y40D12A.2* qPCR AS | TCAACGTAGTTGCAAAGCGT | PYNS572 |
| *F41C3.5* qPCR S | AGAGGAGTTGCTCCAGCAAT | PYNS551 |
| *F41C3.5* qPCR AS | AGCATCTCAGTATCGTTGAG | PYNS552 |
| *F32A5.3* qPCR S | TTCTGGATATGTCGATGCAA | PYNS602 |
| *F32A5.3* qPCR AS | TCCAAGAGATGAGCATCCTG | PYNS603 |
| *K10B2.2* qPCR S | TACCATGCTTTAGTTCGTGAC | PYNS604 |
| *K10B2.2* qPCR AS | TGCTGTAGATGTCACATGTT | PYNS605 |
| *Y16B4A.2* qPCR S | TGCACAAATGCTTCCACCCA | PYNS606 |
| *Y16B4A.2* qPCR AS | ATGCAGCTTGGTTTCTGTAG | PYNS607 |
| *K10C2.1* qPCR S | ATTCACTCCAACGTTCAAGC | PYNS608 |
| *K10C2.1* qPCR AS | TCCTAGAAGTGATGAGCATC | PYNS609 |
| *ctsd-1(asp-1)* qPCR S | GGTGCCAACGCACAAGACCG | PYNS399 |
| *ctsd-1(asp-1)* qPCR AS | AGCCTGGGTCTTGCATGCGG | PYNS400 |
| *asp-3* qPCR S | TGCACAACAGATTTGATTGC | PYNS579 |
| *asp-3* qPCR AS | AGACAACATCGTTGTCAACG | PYNS580 |
| *asp-4* qPCR S | GGTTTCACAGGGTGGAAAGA | PDFZ955 |
| *asp-4* qPCR AS | AGCTTGAGCAAATCCGACAC | PDFZ956 |
| *asp-8* qPCR S | TGGAGTTACTCTGTGGATGGA | PYNS557 |
| *asp-8* qPCR AS | AGTGTATGCTTGATGATTCC | PYNS558 |
| *asp-13* qPCR S | CTAATTGCACAACGTACTGT | PQQZ156 |
| *asp-13* qPCR AS | AATAGCGAGTTGTGCTTCTC | PQQZ157 |
| *ctsb-1* qPCR S | CGCCAAGGACAAGCACTTCGGA | PYNS397 |
| *ctsb-1* qPCR AS | ACCTTGGCCTTTCCGGCGAC | PYNS398 |
| *cpr-2* qPCR S | ACCTTCAAACTCCACCGTGT | PDFZ1007 |
| *cpr-2* qPCR AS | AATAGCAGCGACGGTTCTTG | PDFZ1008 |
| *cpr-3* qPCR S | AAGAATTGCCCGGAGTCAAC | PYNS427 |
| *cpr-3* qPCR AS | AGTTTGAATTTCGGTGACGGA | PYNS428 |
| *cpr-4* qPCR S | ATGAAATACCTCATTCTTGC | PQQZ122 |
| *cpr-4* qPCR AS | CTCAGCCTTCCAGAGAGATT | PQQZ123 |
| *cpr-5* qPCR S | TGTGTCGACTCCTGCACTTC | PDFZ977 |
| *cpr-5* qPCR AS | TGGATTTGCTCGACCTTCTT | PDFZ978 |
| *cpr-6* qPCR S | TGAGATCGCTTTCGAGGTTT | PDFZ953 |
| *cpr-6* qPCR AS | CCAGTCGGTGTTCCAAGAGT | PDFZ954 |
| *cpr-8* qPCR S | TCCTGTGGGGAAGGGTGTGAA | PYNS567 |
| *cpr-8* qPCR AS | TGTACACGCTGGGTAGGTCA | PYNS568 |
| *T28H10.3* qPCR S | TTCTTGAAACAAACGACAAT | PQQZ164 |
| *T28H10.3* qPCR AS | AGTCAACTGTGAGTACTTCT | PQQZ165 |
| *cpl-1* qPCR S | TGGCTCCATTCAATGTTCAG | PYNS338 |
| *cpl-1* qPCR AS | TGTTGTCCTTCGAGGGCTCCGG | PYNS339 |
| *cpz-1* qPCR S | ACGTTACGATAGAATCTACGA | PYNS433 |
| *cpz-1* qPCR AS | AATGCCCAGCATGAGCCGCAAT | PYNS434 |
| *tag-196* qPCR S | ACATTGGTCAAAACCGATTG | PYNS429 |
| *tag-196* qPCR AS | AGACCATAACCATGTCTTTT | PYNS430 |
| *tag-329* qPCR S | AGGAATAAGAAAGTTGGTGG | PYNS431 |
| *tag-329* qPCR AS | ATTGCTTTCTTTAAGTGCACT | PYNS432 |
| *Y40H7A.10* qPCR S | TCTCCAGGAATTTGGATTTG | PYNS555 |
| *Y40H7A.10* qPCR AS | CATTAAGTACTTTTTCCATTC | PYNS556 |
| *F15D4.4* qPCR S | TTGAGGATAAAGTCCGAAAG | PYNS553 |
| *F15D4.4* qPCR AS | ATCATCGGTGAAACCGACAA | PYNS554 |
| *lipl-1* qPCR S | TCATGCGATGGGGATATCCT | PYNS352 |
| *lipl-1* qPCR AS | TCAAGACCGTGTTGCATGAA | PYNS353 |
| *lipl-2* qPCR S | TGCGCACAAGTTCTCTCCCGA | PYNS358 |
| *lipl-2* qPCR AS | AGAGTTCAGCCTCTTTTTCT | PYNS359 |
| *lipl-3* qPCR S | ACAGATGACGGGTACATTTT | PYNS354 |
| *lipl-3* qPCR AS | TGCTCGGGCAGGTTCATAGT | PYNS355 |
| *lipl-4* qPCR S | ACTGTCAAATTACTCAAAAAGTG | PYNS437 |
| *lipl-4* qPCR AS | TCCAGAATGAAGCCATCTTT | PYNS438 |
| *lipl-5* qPCR S | ATGATCTACACAGTTGCCAC | PYNS356 |
| *lipl-5* qPCR AS | TCACGACCCAGTCGCTAGAG | PYNS357 |
| *lipl-6* qPCR S | AATGAGAGCAAGAAAGGAAA | PYNS594 |
| *lipl-6* qPCR AS | TTCGTAACCTCATCATCGTT | PYNS595 |
| *lipl-7* qPCR S | AAGATATTCGAGACTCGATT | PQQZ138 |
| *lipl-7* qPCR AS | CTTGGATAGTTTCGATGATT | PQQZ139 |
| *lipl-8* qPCR S | TGATTGCTTACGGGCAGAAG | PYNS596 |
| *lipl-8* qPCR AS | TTCCCGTACATCAACTTCATC | PYNS597 |
| *asm-1* qPCR S | ACGATTCTGGCCAACATGGT | PYNS514 |
| *asm-1* qPCR AS | AAGTCCATCCATAACTTGTG | PYNS515 |
| *asm-2* qPCR S | CAATCTATGTTCCCGCAGGT | PDFZ971 |
| *asm-2* qPCR AS | AATCATGCGCCTCAAAGTCT | PDFZ972 |
| *asm-3* qPCR S | TGACCGACCTTCATGTTGAC | PYNS383 |
| *asm-3* qPCR AS | AAGGTCCAGCAGGTTGTTTG | PYNS384 |
| *sul-1* qPCR S | TGACCAGGATATTGAACTTGGA | PDFZ945 |
| *sul-1* qPCR AS | ACTGGGACAGCAGATTGGAG | PDFZ946 |
| *sul-2* qPCR S | AAATCGTCGCTGGTCAAAGT | PDFZ947 |
| *sul-2* qPCR AS | CAAATTCTGTGGGCCTTGAT | PDFZ948 |
| *sul-3* qPCR S | CATTGACAATCACGGCTCTG | PDFZ981 |
| *sul-3* qPCR AS | ACAACCGCGTCCTATTTTTG | PDFZ982 |
| *hex-1* qPCR S | GTTGTTCCCATTCCCAGTTG | PDFZ973 |
| *hex-1* qPCR AS | TTCACTGGCTCCGTGTACTG | PDFZ974 |
| *hex-2* qPCR S | AAGAGAATCCATTTGTCAAT | PQQZ128 |
| *hex-2* qPCR AS | TGGTGGAGCACCTTTGAGAT | PQQZ129 |
| *hex-3* qPCR S | ATCGTCACAATAATCTTCCT | PQQZ130 |
| *hex-3* qPCR AS | CTGAGCTTTTGGAGCTTGAA | PQQZ131 |
| *hex-4* qPCR S | TTAGTGAGGATTTGATTGAT | PQQZ132 |
| *hex-4* qPCR AS | CATTCGCTCTATACATCGTT | PQQZ133 |
| *hex-5* qPCR S | GCCTACGAAAGAACGCTGAA | PQQZ134 |
| *hex-5* qPCR AS | TATCAATGTTGTCGACTGAC | PQQZ135 |
| *gba-1* qPCR S | TTCGGATACAGGACTTAACC | PQQZ114 |
| *gba-1* qPCR AS | TCCACTGGAAATCTTCCTTA | PQQZ115 |
| *gba-2* qPCR S | TTCCCCGCATTGGCTCAATT | PYNS598 |
| *gba-2* qPCR AS | TGCACTTCGTTCAATTTCTGG | PYNS599 |
| *gba-3* qPCR S | TGCTGTTCTTCTTGATATTACTC | PYNS423 |
| *gba-3* qPCR AS | TGCATAACTTTCTGCGCGGAA | PYNS424 |
| *gba-4* qPCR S | TCTGGCGTTTTATGGATTTTC | PYNS561 |
| *gba-4* qPCR AS | TCCCAGAAGTAACAGTTCCA | PYNS562 |
| *pho-1* qPCR S | ATGACACTGATCATTTGGGT | PYNS563 |
| *pho-1* qPCR AS | TCGTAAGATTTCCTAGCACT | PYNS564 |
| *pho-10* qPCR S | ACGCATCAAAGGAGGTATAT | PQQZ176 |
| *pho-10* qPCR AS | TGGGGTGAATGGATAGTCAACAT | PQQZ178 |
| *Y105E8B.9* qPCR S | ATACTCTGTCATGGTCAACA | PYNS443 |
| *Y105E8B.9* qPCR AS | AGTCAAAGTTTCCGACGTTC | PYNS444 |
| *rnst-2* qPCR S | CGATGTGGCTGGAGGATTG | PYBL133 |
| *rnst-2* qPCR AS | GCGAATGTCACCGAGAAGG | PYBL134 |
| *M05B5.4* qPCR S | CCAAGCAGACAGCCGATTAT | PDFZ959 |
| *M05B5.4* qPCR AS | CAACTCCTGGCATGTTTTCA | PDFZ960 |
| *vha-1* qPCR S | ATGTCGACCGACACTAAACA | PYNS460 |
| *vha-1* qPCR AS | AAGCGATTCCGGTGCCGGCT | PYNS461 |
| *vha-2* qPCR S | TCATCATGAAATCGGTCATTCC | PYNS458 |
| *vha-2* qPCR AS | AGTCCAGCTGCCAAGTGAGC | PYNS459 |
| *vha-3* qPCR S | ATGTCGTACGACTTGGAAAC | PYNS456 |
| *vha-3* qPCR AS | TGGCGGTTCCGTAGGCGGCT | PYNS457 |
| *vha-4* qPCR S | ATGGCTTTCGTGTTCGTCGGA | PYNS466 |
| *vha-4* qPCR AS | TGAGACCACCTCCAAAGATC | PYNS467 |
| *vha-5* qPCR S | AGGATGCTGCGTTCAACATT | PYNS387 |
| *vha-5* qPCR AS | ATCTCATCGTAACGACGGAT | PYNS388 |
| *vha-6* qPCR S | AGAATGCGTACACGAGAAAA | PYNS464 |
| *vha-6* qPCR AS | ATGCTCATCGTAGTCTGGAA | PYNS465 |
| *vha-7* qPCR S | ACGATCGGCGTGAGCATATT | PYNS385 |
| *vha-7* qPCR AS | ACGATCTTCGATGGATTTGT | PYNS386 |
| *vha-8* qPCR S | ACGCAAAATTCAAGCCTCCA | PYNS462 |
| *vha-8* qPCR AS | ATAACGAGCAGCATCTCCGGA | PYNS463 |
| *vha-9* qPCR S | TTATCAATCAGCACATCGCCGA | PYNS452 |
| *vha-9* qPCR AS | AACAATCCACGGGCTCTGTT | PYNS453 |
| *vha-10* qPCR S | AATCTGTGGCTGGAAACAAG | PYNS454 |
| *vha-10* qPCR AS | TTAGGCAGCGAACTGTCCGT | PYNS455 |
| *vha-11* qPCR S | AGTTATTCGCAAACTTGTGC | PYNS405 |
| *vha-11* qPCR AS | ATTGGAATTTCGTCACGTAC | PYNS406 |
| *vha-12* qPCR S | TCTCAAGCTCAATATCGTGT | PYNS450 |
| *vha-12* qPCR AS | TTGGCGTCAATTCCGGAGGT | PYNS451 |
| *vha-13* qPCR S | TCTGATGCTATTATTTACGTCG | PYNS481 |
| *vha-13* qPCR AS | TGTACGCTTCATGATGGAAG | PYNS482 |
| *vha-14* qPCR S | TGGCTCCTCCCTTTCCGAGA | PYNS449 |
| *vha-14* qPCR AS | TCTCAAGCTCAATATCGTGT | PYNS450 |
| *vha-15* qPCR S | GGGGATCTTACTTCCGCTCT | PDFZ923 |
| *vha-15* qPCR AS | CCATGGTCTTAGCTGCCTGT | PDFZ924 |
| *vha-16* qPCR S | ATCCACTTGGATCATTCGAG | PYNS474 |
| *vha-16* qPCR AS | ATCTCGTCCAAATCTTGCTC | PYNS475 |
| *vha-17* qPCR S | TTATGACTGCTGTTTGCTGC | PYNS403 |
| *vha-17* qPCR AS | AGAAATCCAGCGAATTGTTT | PYNS404 |
| *vha-18* qPCR S | AGAGTAACGATCCATTAGTG | PYNS470 |
| *vha-18* qPCR AS | TGCTTCTTTACCACCGAGTT | PYNS471 |
| *vha-19* qPCR S | ATCGGCCGATTACTTGGCAA | PYNS477 |
| *vha-19* qPCR AS | TCCGTAGATAATGTAGATTG | PYNS478 |
| *unc-32* qPCR S | ATCAGATCCCGATGCTGGAT | PYNS468 |
| *unc-32* qPCR AS | TTCAGCGTTTCCTCGTTCTT | PYNS469 |
